# Characteristics of Glycerolized Chitosan: NH_4_NO_3_-Based Polymer Electrolyte for Energy Storage Devices with Extremely High Specific Capacitance and Energy Density Over 1000 Cycles

**DOI:** 10.3390/polym12112718

**Published:** 2020-11-17

**Authors:** Shujahadeen B. Aziz, M. A. Brza, Iver Brevik, M. H. Hamsan, Rebar T. Abdulwahid, S. R. Majid, M. F. Z. Kadir, Sarkawt A. Hussen, Ranjdar M. Abdullah

**Affiliations:** 1 Hameed Majid Advanced Polymeric Materials Research Lab., Physics, College of Science, University of Sulaimani, Qlyasan Street, Kurdistan Regional Government, Sulaimani 46001, Iraq; rebar.abdulwahid@univsul.edu.iq (R.T.A.); sarkawt.hussen@univsul.edu.iq (S.A.H.); ranjdar.abdullah@univsul.edu.iq (R.M.A.); 2 Department of Civil engineering, College of Engineering, Komar University of Science and Technology, Kurdistan Regional Government, Sulaimani 46001, Iraq; 3 Manufacturing and Materials Engineering Department, Faculty of Engineering, International Islamic University of Malaysia, Gombak, Kuala Lumpur 50603, Malaysia; mohamad.brza@gmail.com; 4 Department of Energy and Process Engineering, Norwegian University of Science and Technology, N-7491 Trondheim, Norway; 5 Centre for Foundation Studies in Science, University of Malaya, Kuala Lumpur 50603, Malaysia; hafizhamsan93@gmail.com (M.H.H.); mfzkadir@um.edu.my (M.F.Z.K.); 6 Centre for Ionics, Department of Physics, Faculty of Science, University of Malaya, Kuala Lumpur 50603, Malaysia; shana@um.edu.my

**Keywords:** biopolymer electrolyte, XRD study, impedance spectroscopy, ion transport parameters, TNM and LSV measurement, energy storage, EDLC device

## Abstract

In this work, plasticized polymer electrolyte films consisting of chitosan, ammonium nitrate (NH_4_NO_3_) and glycerol for utilization in energy storage devices was presented. Various microscopic, spectroscopic and electrochemical techniques were used to characterize the concerned electrolyte and the electrical double-layer capacitor (EDLC) assembly. The nature of complexation between the polymer electrolyte components was examined via X-ray diffraction analysis. In the morphological study, field emission scanning electron microscopy (FESEM) was used to investigate the impact of glycerol as a plasticizer on the morphology of films. The polymer electrolyte (conducting membrane) was found to have a conductivity of 3.21 × 10^−3^ S/cm. It is indicated that the number density (*n*), mobility (*μ*) and diffusion coefficient (*D*) of ions are increased with the glycerol amount. The mechanism of charge storing was clarified, which implies a non-Faradaic process. The voltage window of the polymer electrolyte is 2.32 V. It was proved that the ion is responsible for charge-carrying via measuring the transference number (TNM). It was also determined that the internal resistance of the EDLC assembly lay between 39 and 50 Ω. The parameters associated with the EDLC assembly are of great importance and the specific capacitance (*C_spe_*) was determined to be almost constant over 1 to 1000 cycles with an average of 124 F/g. Other decisive parameters were found: energy density (18 Wh/kg) and power density (2700 W/kg).

## 1. Introduction

Various resources of electrical energy are accounted worldwide: 42% from coal, 21% from natural gas, 15% from hydro, 14% from nuclear, 5% from oil and 3% from renewable energy devices [1]. The decay of natural gas and oil is estimated to reach a maximum over the next few decades [2]. Moreover, the problem of energy obtained from coal is the emission of CO_2_ into the air, resulting in global warming [3].

To avoid these problems, the development of renewable energy devices, such as electrochemical capacitors, namely supercapacitors (SCs) and ultracapacitors, has been focused on by several research groups [4,5,6,7]. SC is a general term for electrical double-layer capacitors (EDLCs), pseudocapacitors (PCs) or hybrid capacitors. They are characterized by their fast charging process, relatively high power density, excellent cyclability and ease of fabrication [8,9,10]. EDLCs are considered as a potential alternative to conventional batteries. The principle of EDLCs is based on the non-Faradaic process that energy is stored in the form of charge accumulation at the interfacial region between the carbon-based electrode and electrolyte [11,12,13].

In comparison, EDLCs are characterized by relatively high power density, electrochemical and thermal stabilities, plausible reversibility, cheapness, safety and simplicity of fabrication, which is superior to Faradaic capacitors (PCs) [14,15,16,17]. As stated previously, EDLCs are superior over other energy devices in terms of utilizing carbon materials as inexpensive materials in the fabrication of electrodes [18]. The desired property of the activated carbon (AC) materials is micro-porosity, which provides a high surface area to expose to the interfacial region to encompass as many ions as possible. For example, it was documented that the high specific area of activated carbon ranged from 1879 to 3313 m^2^/g [19]. To enrich the interfacial region with ions, inorganic acids, such as sulfuric acid (H_2_SO_4_) and phosphoric acid (H_3_PO_4_), have been widely used. Nowadays, ammonium salts have been used as a strong alternative to inorganic acids. This is owing to the chemical degradation of these inorganic acids in EDLC devices [20].

Biodegradable polymer electrolytes are considered as a good candidate to be employed in energy devices such as EDLCs and reduce electronic waste. These materials are essential for applications as ionic conductive and flexible membranes [21,22,23]. Polymer electrolyte is superior to liquid electrolytes, which possess better mechanical properties, ease formation of thin film, are an appropriate size and have appropriate contact between the electrode and the electrolyte [24,25]. It is interesting to notice that the presence of electronegative atoms, for instance, oxygen and nitrogen, within the monomer of the chain of polymers enhances the solvent properties to dissolve salts and then to provide ions. Fortunately, there are oxygen and nitrogen in chitosan (CS), which thereby encourages researchers to focus on this fascinating polymer material [26,27,28]. Consequently, it is possible to synthesize polymer electrolytes by dissolving metal salts in polymeric matrices. This methodology makes polymers conductive ionically, which has intensive applications in electrochemical devices [29,30].

The main focus is devoted to utilizing natural polymers as environmentally friendly and impressive characteristic solid polymer electrolytes in electrochemical devices, such as electrochromic devices, sensors and fuel cells [31,32]. In addition, recently, polymer electrolytes have been widely employed in batteries, which can have both economic and environmental benefits [33,34]. Herein, some examples of biodegradable and biocompatible natural polymers in polymer electrolytes are given [35]. The most abundant and utilized one is chitosan (CS), which is the deacetylated product (i.e., a derivative) of chitin, in addition to cellulose [36,37]. The main natural source of CS is shrimp waste, in which extraction has been performed extensively [38]. In addition to all these superior properties of CS, there is the enrichment of structure with polar groups, such as NH_2_ and OH. These polar groups serve as conjunction sites, providing an association with some transition metal ions [39,40]. Another attractive property of CS is the capacity for molding into a range of forms, such as hydrogels and porous scaffolds [41].

The present study is aimed at preparing polymer electrolyte using biodegradable host polymer (CS) with ammonium nitrate (NH_4_NO_3_) as a source of ions. The salt possesses relatively low lattice energy of 642 kJ/mol, which means a high degree of dissociation [42]. To further enhance conductivity, CS:NH_4_NO_3_: glycerol has also been used as a relatively high conducting electrolyte between the electrodes in the fabricated EDLC.

## 2. Materials and Methods

### 2.1. Sample Preparation

The raw materials, CS (average molecular mass: 310,000–375,000) and ammonium nitrate (NH_4_NO_3_), were purchased from Sigma-Aldrich (St. Louis, MO, USA). To prepare the polymer electrolytes, 1 g of CS was dissolved in 60 mL of 1% acetic acid with stirring for 3 h until a homogeneous solution was gained at ambient temperature. In the preparation of CS:NH_4_NO_3_ electrolytes, 40 wt.% of NH_4_NO_3_ was added into the CS solution with vigorous stirring. Afterwards, the coding process of prepared polymer electrolyte was performed in a way CSNHG1, CSNHG2, CSNHG3 and CSNHG4 for CS:NH_4_NO_3_ plasticized with 10, 20, 30 and 40 wt.% of glycerol, respectively. After leaving these polymer electrolytes, dry films formed at ambient temperature. Then, casting in Petri dishes was carried out, and to produce the solvent-free film, the films were put into a desiccator. All these preparations were conducted under the conditions of ~50% and relative humidity at 25 °C.

### 2.2. X-ray Diffraction (XRD) Study

It is essential to know about the nature of the complexation between electrolyte components using XRD. The patterns of XRD were acquired using an X-ray diffractometer (Bruker AXS, Karlsruhe, Germany) with operating voltage and current of 40 kV and 40 mA, respectively, at ambient temperature. The scanning over the samples was carried out using a monochromatic beam (X-radiation of wavelength (λ = 1.5406 Å) at glancing angles in the range of 5° ≤ 2θ ≤ 80° with a step size of 0.1°. The degree of crystallinity (*X_c_*) was calculated using the following equation [43].

(1) $$X_{c}=\left[\frac{A_{c}}{\left(A_{c}+A_{a}\right)}\right]\times 100\%$$

where *A_c_* and *A_a_* are areas under the peaks of crystalline and amorphous peaks, respectively.

### 2.3. Field Emission Scanning Electron Microscopy (FESEM)

The surface images of the produced dry films were obtained with a field-emission microscope, using a Hitachi SU8220 (Hitachi, Tokyo, Japan) at 500× magnifications.

### 2.4. Electrochemical Impedance Spectroscopy (EIS)

Complex impedance spectroscopy as a powerful technique is not only for determining the electrical properties of materials but also for investigating the interfacial region between the electronically conducting electrodes and the electrolytes. As described in the experimental section, the separating phase was obtained from the solid polymer electrolyte (SPE) films by cutting them into small discs (2 cm diameter) and sandwiching them between two stainless steel electrodes using spring pressure. The impedance of the films was measured using a HIOKI 3531 Z Hi-tester (HIOKI, Nagano, Japan), which was controlled by a computer in the frequency range from 50 Hz to 5000 kHz. Software was used to measure the real (*Z_r_*) and imaginary (*Z_i_*) parts of impedance. To present the impedance spectra of *Z_r_* and *Z_i_*, a Nyquist plot was used, and an analysis of the spectra was performed from the determination of the intercept of the plot with the real impedance axis to gain the bulk resistance. All conductivity values were also calculated using the equation shown below [24,25].

(2) $$\sigma_{dc}=\left(\frac{1}{R_{b}}\right)\times \left(\frac{t}{A}\right)$$

where *t* and *A* are the thickness and surface area of the film, respectively.

### 2.5. Transference Number Measurement (TNM)

Both ion (*t_ion_*) and electron (*t_el_*) transference numbers were measured from cell polarization of stainless steel (SS) | relatively high conducting SPE | SS by holding the voltage at 0.20 V. A V&A Instrument DP3003 digital *DC* power supply (V & A Instrument, Shanghai, China) was used in the data acquisitions. The ion and electron transfer numbers of the films were obtained using the equations shown below [25,26,27].

(3) $$t_{ion}=\frac{I_{i}-I_{ss}}{I_{i}}$$

(4) $$t_{el}=1-t_{ion}$$

where *I_i_* and *I_ss_* are the initial and the steady-state currents, respectively.

### 2.6. Linear Sweep Voltammetry (LSV) 

The electrochemical stability of the SPE system was determined using linear sweep voltammetry (LSV) at ambient temperature. The cell design for recording LSV consisted of SS | relatively high conducting SPE | SS. A Digi-IVY DY2300 potentiostat (Neware, Shenzhen, China) was used for recording the LSV at a sweep rate of 10 mV/s and at ambient temperature.

### 2.7. EDLC Fabrication

The dry mixing process was used in constructing electrodes for EDLC by adding 0.25 g of carbon black to 3.25 g of activated carbon in a planetary ball miller. A solution of polyvinylidene fluoride (PVdF) was prepared by dissolving 0.50 g in 15 mL N-methyl pyrrolidone (NMP) at ambient temperature. All materials were purchased from Sigma-Aldritch (St. Louis, MO, USA). Afterwards, the dry mixture of activated carbon-carbon black powders was dispersed in PVdF-NMP solution for 90 min. Then, stirring of the mixture for a few hours was carried out until a thick black solution was obtained. The current collector (aluminium foil) was coated with a black solution with the aid of a doctor blade followed by drying of the coated aluminium foil in an oven at 60 °C. To ensure the dryness of these electrodes and maintain a geometric surface area of 2.01 cm^2^ and thickness of ~0.02 cm, a desiccator containing silica gel was used. The cell design of EDLC was as follows: AC electrode | relatively high conducting SPE | AC electrode. This designed cell was packed in CR2032 coin, and all electrochemical measurements of the fabricated EDLC were performed using a Digi-IVY DY2300 potentiostat (10 mV/s sweep rate).

## 3. Results and Discussion

### 3.1. X-ray Diffraction (XRD)

**Figure 1:**
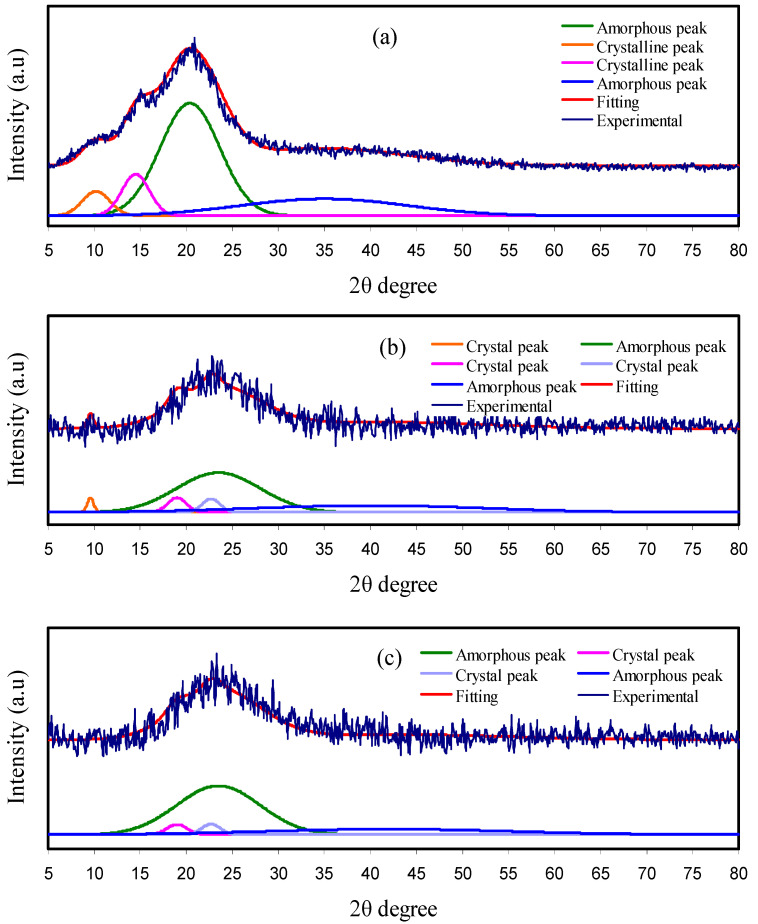
X-ray diffraction (XRD) spectra for (**a**) pure chitosan (CS), (**b**) CSNHG2 and (**c**) CSNHG4 electrolyte films.

The XRD spectra of the plasticized CS-based electrolyte films are exhibited in Figure 1. Previously, it was proved for pure CS that there were many crystalline peaks, in addition to the broad, amorphous peak centres at 2θ ranges from 33° to 45° [24,39]. The XRD spectrum for pure CS is shown in Figure 1a. The crystalline peak centres at 2θ = 11.2°, which can be related to the (0 2 0) reflection plane, and one around 20° can be ascribed to the contribution of two peaks as observed in the doped samples at 18.16° and 22.7°. These could be correlated to the reflections of the (2 0 0) and (2 2 0) planes. Moreover, another peak appearing near 15° has already been recorded to be a demonstration of the existence of relatively regular crystal lattice (110) of CS [44]. Figure 1b,c shows the XRD pattern for CS:NH_4_NO_3_ electrolytes incorporated with 20 wt.%, and 40 wt.% glycerol. The appearance of peaks for pure CS around 2θ = 15° and 20° are seen as distorted in the form of broad humps in the CS:NH_4_NO_3_:glycerol electrolytes. It is seen that the central peak of CS appearing at around 2θ = 20° is distributed over two peaks with a broad linewidth feature. The strong evidence of a developing amorphous phase in the CS:NH_4_NO_3_:glycerol electrolytes upon insertion of glycerol are thus lowering in intensity and broadening of the diffraction peaks as shown in the XRD pattern [39,45,46]. The developing amorphous phase in CS:NH_4_NO_3_:glycerol electrolyte is mainly caused by the complexation between functional groups of the polymer/plasticizer and cation of the salt as a result of electrostatic interactions [25,26]. This finding indicates the effectiveness of glycerol as a plasticizer in lowering the crystalline phase of the CS polymer. In addition, glycerol as aplasticizer is characterized by cheapness, high natural abundance, less toxicity and is environmentally friendly.

Furthermore, with increasing the quantity of glycerol, an amorphous phase increasingly appears that is accompanied by the disappearance of crystalline peaks of CS. Origin 8 software was used to measure the degree of crystallinity (*X_c_*) of the fabricated membranes, using calculation of area under the peaks for both amorphous and crystalline phases. The values of *X_c_* were calculated using Equation (1). It is interesting to notice that the *X_c_* lowers effectively upon the addition of more glycerol, as shown in Table 1. This is associated with the disruption of interaction within the polymer crystalline phase [47]. Herein, the degree of crystallinity of pure CS is 15.97%, which is close to that documented in the literature (15.1%) [48].

The relatively crystalline structure of CS originates from the existence of both intramolecular and intermolecular hydrogen bonding [49,50]. However, the plasticized CS samples were characterized by dominancy of an amorphous phase, as confirmed using XRD. This development in the amorphous phase results in an enhancement in ionic conductivity. Figure 1 confirms the abundance of the amorphous nature of CS solid electrolyte samples from these Gaussian-shaped broad peaks, as examined by Alves et al. [51].

### 3.2. Field Emission Scanning Electron Microscopy (FESEM)

**Figure 2:**
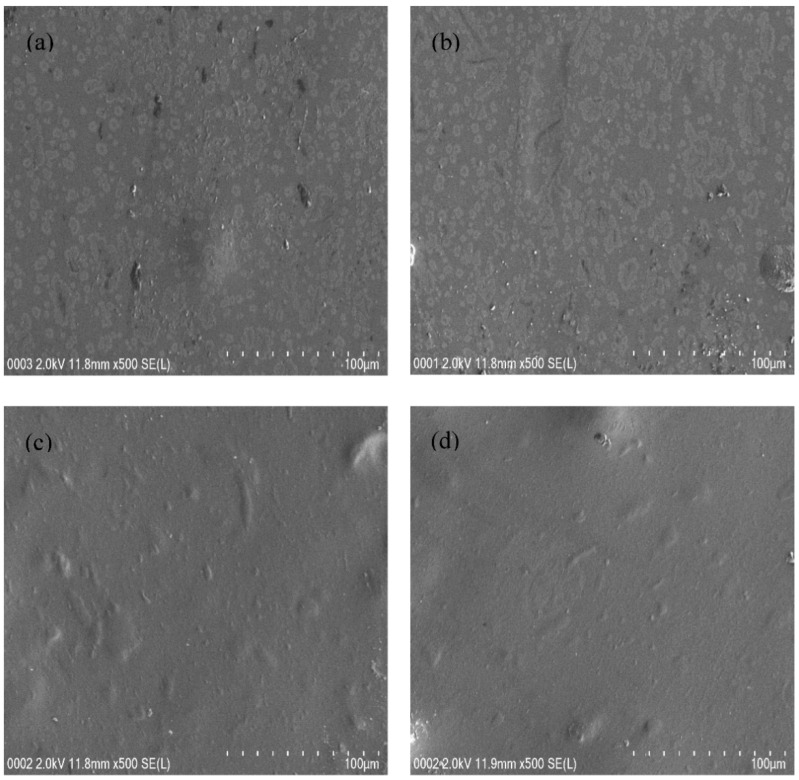
Field emission scanning electron microscopy (FESEM) images for (**a**) CSNHG1, (**b**) CSNHG2, (**c**) CSNHG3, and (**d**) CSNHG4 electrolytes.

Morphological study of the films could support the XRD results. For this purpose, FESEM was used to have surface images of films with 500× magnification. Figure 2a–d show the FESEM of all film samples. A few protrudes over the film were seen with the addition of 10 and 20 wt.% glycerol into the electrolyte system, as shown in Figure 2a,b. It is interesting to notice that there are no protrudes as the glycerol quantity was increased from 20 to 40 wt.%, as presented in Figure 2c,d. In other words, the plasticized electrolyte samples are characterized by surface smoothness and uniformity with the absence of any phase separation. As a consequence, the FESEM and the XRD results support each other. It was documented that the appearance of smooth morphology is associated with the amorphous phase enhancement in the electrolyte samples [50]. The smooth surface electrolytes can facilitate ion conduction, thereby, an increase of *DC* conductivity value was achieved [50].

### 3.3. Impedance and Ion Transport Parameters Study

**Figure 3:**
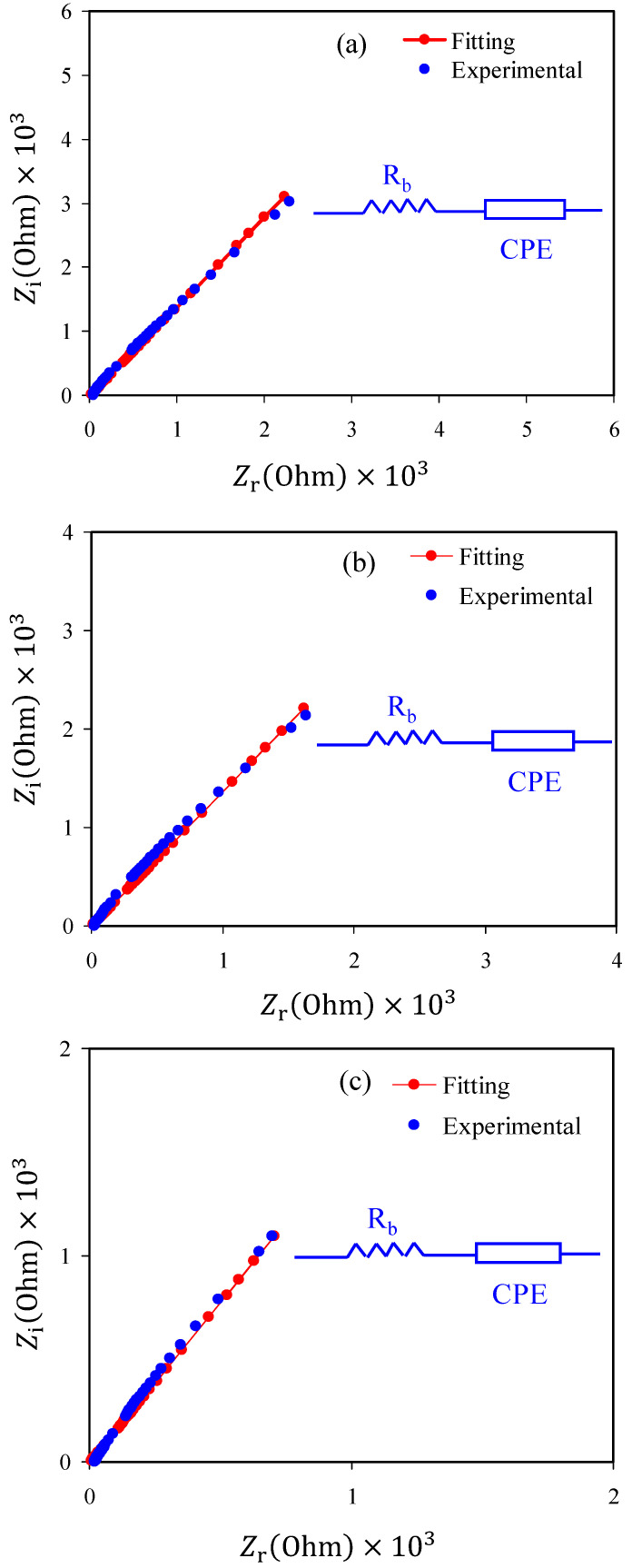

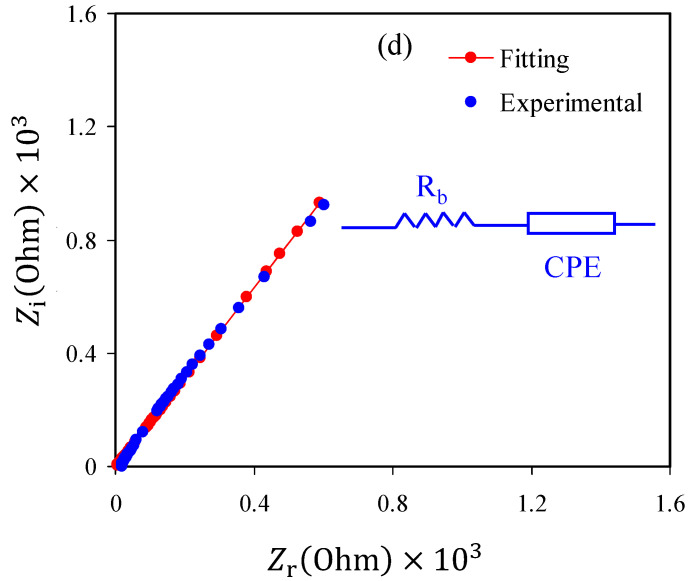
Electrochemical impedance spectroscopy (EIS) plots for (**a**) CSNHG1, (**b**) CSNHG2, (**c**) CSNHG3 and (**d**) CSNHG4 electrolyte films.

In the study of electric properties and mechanism of ion transport and electron transfer in polymer electrolytes and conjugated polymers, it is appropriate to use an impedance spectroscopic technique [52]. The complex impedance Nyquist plots (*Z*′ versus *Z*″) of plasticized CS:NH_4_NO_3_ electrolyte film samples at ambient temperature are shown in Figure 3a–c. It is seen that only a distinct tail exists in the Nyquist plots. In the literature, it is emphasized that the impedance spectra of low ionic conductivity polymer electrolytes are characterized by a half semicircle and a tail at high frequency and low-frequency regions, respectively [15,24,30,45,46]. In the earlier studies, this tail showed that *DC* conductivity values ranged from 10^−4^ to 10^−3^ S/cm [53,54,55] for the polymer electrolytes. At the low-frequency region, the spike would be related to the capacitor obtained as a result of the double-layer formation from charge accumulation at the electrode/electrolyte interfacial region [56,57,58,59]. It is well-defined that the tail at low frequency has deviated from the imaginary axis results from ion moving when the electrode polarization occurs [56,58,60]. From the analysis of the intercept between the spike at the low-frequency region and the real axis in the complex impedance plots, the electrical bulk resistance (*R_b_*) of the samples can be extracted. If the area and thickness of the electrodes and *R_b_* are known, it is straightforward to calculate the *DC* ionic conductivity using Equation (2). The values of *DC* ionic conductivity continuously increase with increasing glycerol content, as presented in Table 2. From Table 2, it is clear that proton (H^+^) ion conductivity was influenced by glycerol concentration. In the present work H^+^ cation is provided by the dissolved NH_4_NO_3_ salt. To investigate the electrical impedance spectroscopy of polymer electrolytes, it is straightforward and rapid to use the electrical equivalent circuit (EEC) model [61,62]. From the Nyquist plots and modelling the electrical circuit (equivalent circuit) of the polymer electrolytes, the *R_b_* for the charge carriers and a constant phase element, i.e., *CPE*, are obtained, as shown in the inset of Figure 3a–c. The EEC fitting parameters are shown in Table 3. The *Z_CPE_* impedance is mathematically expressed as follows [53,54,55,62].

(5) $$Z_{CPE}=\frac{\cos(\pi n/2)}{C\omega^{n}}-j\frac{\sin(\pi n/2)}{C\omega^{n}}$$

where *C* is the *CPE* capacitance and *ω* and *n* are angular frequency and the vertical axis deviation of the plot, respectively, in the complex impedance plots.

It is confirmed from the Nyquist plot of polymer electrolyte that the only the resistive component is evident. The polymer can act as an insulator, and the *CPE* was connected in series with *R_b_*, as shown in the inset of Figure 3a–c. It is worth mentioning that the values of *Z_r_* and *Z_i_* of the EEC can be mathematically expressed as follows [53,54,55]:

(6) $$Z_{r}=R+\frac{\cos(\pi n/2)}{C\omega^{n}}$$

(7) $$Z_{i}=\frac{\sin(\pi n/2)}{C\omega^{n}}$$

As the impedance data composed of a tail only, the ionic transport parameters of the diffusion coefficient (*D*), mobility (*μ*) and number density (*n*) of ions are computed by the given equations [12].

The *D* of the ion carriers of the systems is computed using the relation,

(8) $$D=D_{{}^{\circ}}\exp\{-0.0297{\left[\ln D_{{}^{\circ}}\right]}^{2}-1.4348\ln D_{{}^{\circ}}-14.504\}$$

where

(9) $$D_{{}^{\circ}}\;=\left(\frac{4k^{2}l^{2}}{R_{b}{}^{4}\omega_{\min}{}^{3}}\right)$$

where l is the electrolyte thickness and *ω_min_* is the angular frequency corresponding to the minimum *Z_i_*.

The mobility (*µ*) of the ion carriers is calculated using the relation below.

(10) $$\mu \;=\left(\frac{eD}{K_{b}T}\right)$$

where *T* is the absolute temperature and *k_b_* is the Boltzmann constant. 

Since *DC* conductivity of ions is presented by

(11) $$\sigma_{Dc}=ne\mu ,$$

thus, the number density of ion carriers (*n*) is computed using Equation (11).

Table 4 shows the ion transport parameters and the *ω_min_* values for the electrolyte systems. Based on Table 4, the value of *D* is observed to increase when the glycerol concentration increases from 10 to 40 wt.%. The same trend is shown by *μ,* as seen in Table 4, where *μ* increases. The increase of *μ* and *D* is attributed to the chain flexibility enhancement with the glycerol existence. When the glycerol concentration is increased, the values of *D*, *μ* and *n* are increased, which causes the value of conductivity to increase. This is because the extra glycerol addition dissociates more salts to free ions, thus increasing the number density of ion carriers [12].

### 3.4. Electrochemical Investigations

#### 3.4.1. Transference Number Measurement

It is of vital importance to determine the activity and identity of the charge carrier in the polymer electrolyte samples using transference number measurement (TNM). At a constant voltage of 0.2 V, both ions and electrons contributed to the whole conductivity process in the polymer electrolyte samples. It is essential to know the extent of ionic conductivity in the electrolytes in use in batteries and SCs [32].

**Figure 4:**
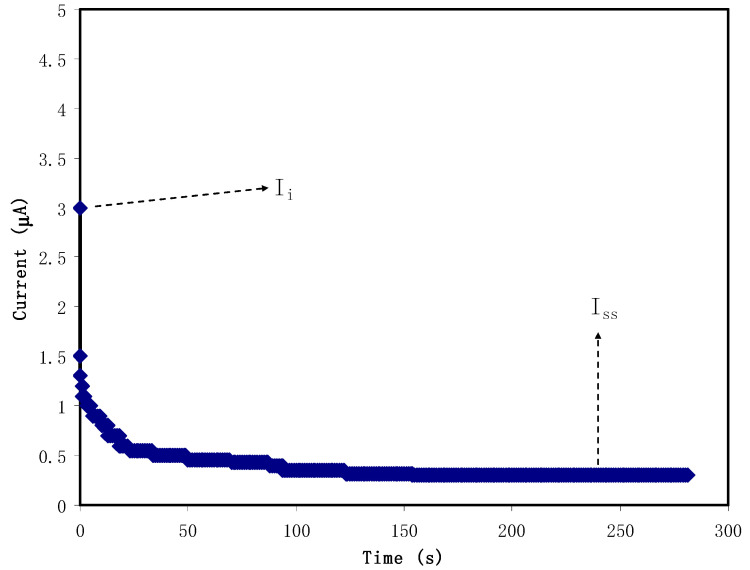
Polarization of the highest conducting electrolyte at 0.2 V working voltage.

Figure 4 shows the polarization response of relatively high conducting CS:NH_4_NO_3_: glycerol electrolyte. It is seen that as the polarization begins, a substantial current (*I_i_*) of 3 µA is recorded resulting from the charge conduction by both electrons and ions. The current then decreases sharply as time proceeds as a result of the decay of the number of ions within the plasticized polymer electrolytes [63]. In this complex electrochemical system, stainless steel (SS) was used as an electrode, which allows electron transfer to pass through and prohibits ion transport (ion-blocking electrode). As a consequence, the current decays until reaching 0.23 µA in the form of a plateau. The steady-state current is one of the electrochemical polarization responses [64]. Herein, to calculate the value of *t_ion_* and *t_el_*, it is straightforward to use Equations (3) and (4). The calculation gives *t_el_* and *t_ion_* values to be 0.077 and 0.923, respectively. Accordingly, the main and primary charge carriers in the plasticized electrolyte are ion as the *t_ion_* is much higher than *t_el_*. Both cations and anions contribute to charge transport within the electrolyte film systems [65].

In EDLC devices, ion accumulation at the interface region is necessary to store charge as a form of energy. Othman et al. reported polymethyl methacrylate (PMMA)-lithium trifluoromethanesulfonate (LiCF_3_SO_3_) with an ion transference number of 0.93 to 0.98 [66]. Two factors govern the value of transference number: ion-ion and ion-polymer interactions. Vijaya et al. recorded a relatively high ion transference number ranging from 0.90 to 0.99 for ammonium salt-based polymer electrolytes [67].

#### 3.4.2. LSV Analysis

**Figure 5:**
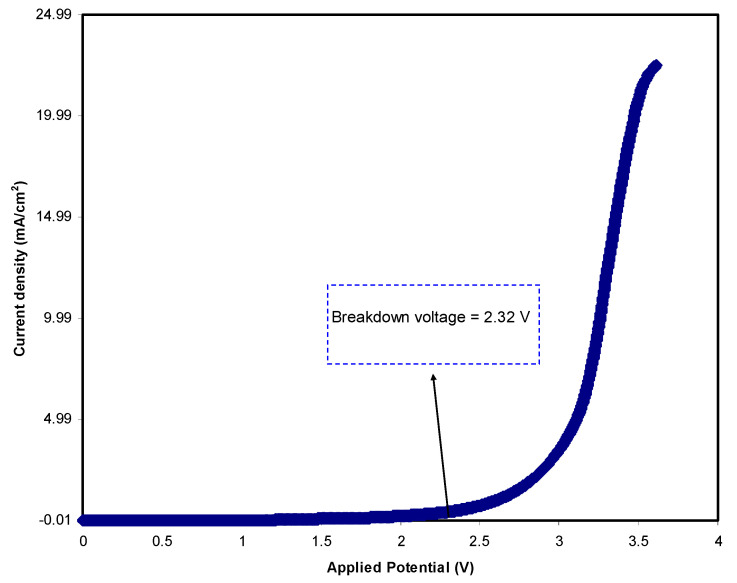
Linear sweep voltammetry (LSV) plot of the highest conducting electrolyte at 10 mV/s.

To determine the electrochemical potential window of the electrolyte, it is informative to record linear sweep voltammetry (LSV). Figure 5 shows the LSV response of the relatively high conducting (CSNHG4) electrolyte at 10 mV/s. It is seen that below 2.32 V, there is no detectable current, indicating a non-electrochemical reaction within this potential range. However, beyond 2.32 V, the polymer electrolyte begins to decompose, showing an electrochemical reaction as a result of the polymer electrolyte decomposition [68]. Monisha et al. have stated that as current passes through a cell system, the voltage at the current value is called the threshold voltage [69]. In a previous study, it was documented that the lithium salt possesses a decomposition voltage of 2.10 V [70]. In the present study, the recorded voltage window for the polymer electrolyte under study is acceptable for utilization in energy storage devices.

### 3.5. EDLC Studies

#### 3.5.1. Cyclic Voltammetry (CV)

**Figure 6:**
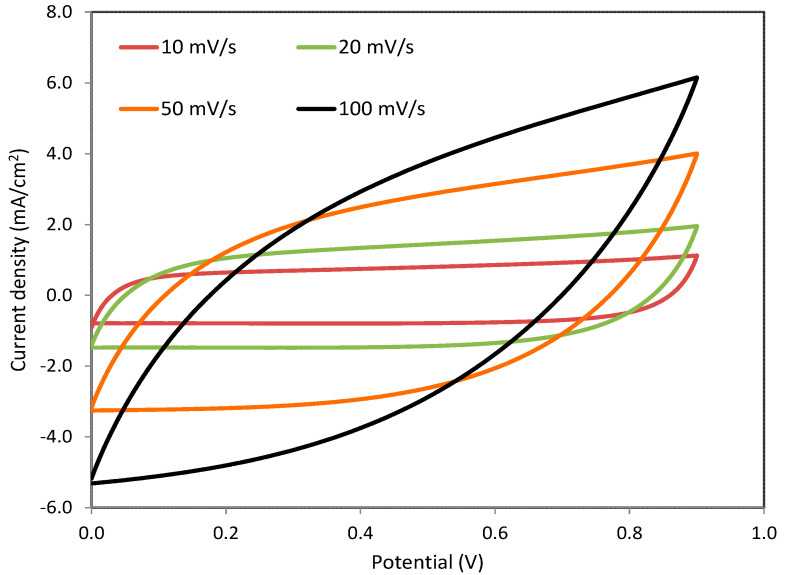
Cyclic voltammetry responses of the relatively high conducting electrolyte at various sweep rates.

Cyclic voltammetry as a straightforward technique was used in the estimation of the capacitance of the EDLC. The voltage window of the CV scan was in the range of 0 and 9 V at various sweep rates: 10, 20, 50 and 100 mV/s. The CV responses of the EDLC are shown in Figure 6. It is seen that there is no oxidation/reduction peak other than the adsorption of cation and anion of NH_4_NO_3_ salt, producing intercalation/deintercalation at the surface of the carbonic electrodes [26,27]. This means that a double layer formed as a result of ion accumulation at the interfacial region between the electrodes and electrolyte, producing capacitive current (non-Faradaic reaction) [71]. Moreover, the main feature of the CV response is a rectangular shape at relatively low sweep rates. The carbon porosity of the electrodes results in relatively high internal resistance, which in turn results in the CV appearing in a leaf-like form [53,54,55].

Moreover, the role of the electron is ignored due to the nonexistence of the redox peak. This suggests the occurrence of a non-Faradaic process, which is desired for the design of EDLCs [72]. The porous feature of the activated carbon and internal resistance facilitates double-layer formation [73]. From the CV, one can estimate the value of the specific capacitance (*C_spe_*) of the EDLC assembly using the relationship shown below [53,54,55].

(12) $$C_{spe}=\frac{\int_{V_{1}}^{V_{2}}I\left(V\right)dV}{2m\left(V_{2}-V_{1}\right)\left(\frac{dV}{dt}\right)}$$

where $\int_{V_{1}}^{V_{2}}I\left(V\right)dV$ is the area under *CV* response using Origin Pro 8.5 software and *m* is the mass of active material (activated carbon), (*V*_2_ − *V*_1_) is the voltage range and $\frac{dV}{dt}$ is the sweep rate. Table 5 presents the *C_spe_* of the EDLC assembly obtained from *CV* responses. In the next section, the *C_spe_* obtained from the *CV* response will be compared to that from the charge-discharge curve. It is interesting to compare the shape of the *CV* of the present work to that of biopolymer-based EDLC studies, which are relatively close to each other [74,75,76].

#### 3.5.2. Galvanostatic Charge-Discharge Study

**Figure 7:**
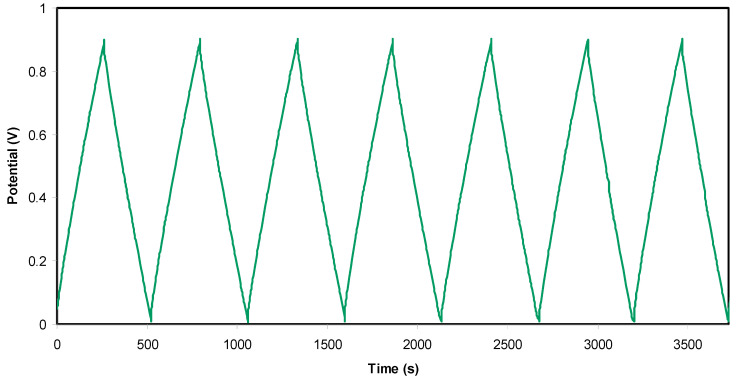
Charge-discharge plot of the electrical double-layer capacitor (EDLC) at selected cycles.

Figure 7 shows the charge-discharge response of the EDLC assembly by holding the current density at 0.5 mA cm^−2^. It is seen that the slope of the discharge side response is almost linear, confirming the capacitive behaviour of the EDLC assembly to a large extent [77]. From the slope value *x* of the discharge side, one can calculate the *C_spe_* of the EDLC assembly using the following relationship:

(13) $$C_{spe}=\frac{i}{xm}$$

where *i* is the applied current. 

**Figure 8:**
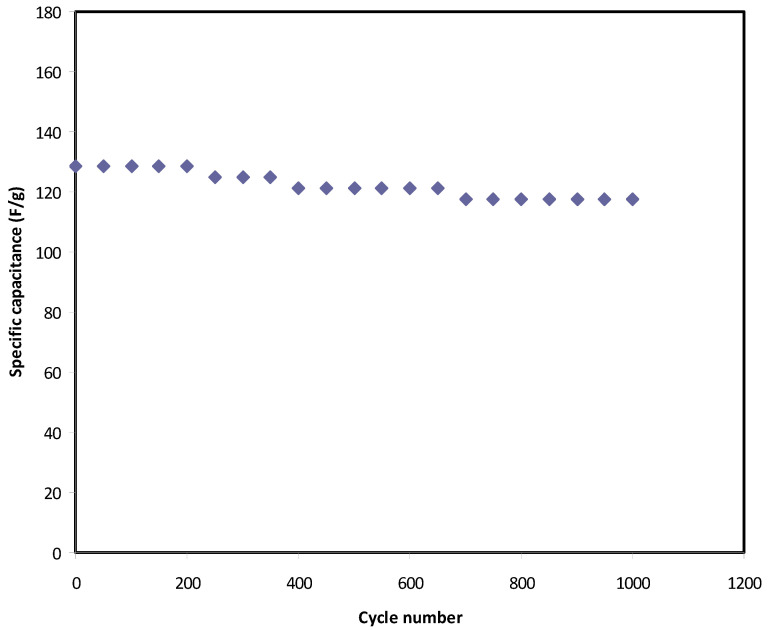
S pecific capacitance of the EDLC throughout the 1000 cycles.

Figure 8 shows the fluctuation of *C_spe_* values over a wide cycle number of the charging-discharging process. At the 1st cycle, the *C_spe_* value was calculated and found to be 128 F g^−1^, which was quite close to that obtained from CV analysis at 10 mV/s. From this result, it has been concluded that the charging-discharging response is reliable in the estimation of *C_spe_* of the EDLC assembly. At the 400th cycle, the *C_spe_* lowers to 120 F g^−1^ and keeps a constant average value of 119 F g^−1^ up to the 1000th cycle. It is well established that the value of specific capacitance refers to the amount electrical charge stored at the interfacial region due to the electrostatic interaction between the electron in the electrode and ions in the electrolyte in the presence of an applied electric field. Due to the rapid charge-discharge process the number of free ions reduced (resulting from ion aggregation and ion pair formation), and thus the ion adsorption process is also affected. These will reduce the electrostatic interaction between ions and electrons at the interfacial region and lead to a little charge storage, which in turn causes a decrease in the value of specific capacitance. In the present study, the value of *C_spe_* is much greater than that documented (61.7 F g^−1^) for an ionic liquid-based gel polymer electrolyte reported by M. Tripathi and S.K. Tripathi [78]. Importantly, the specific capacitance is also greater than that recorded for gel-based polymer electrolytes by Boonen et al. [79] and by Łatoszyńska et al. [80], which were 87.3 F/g and 90 F/g, respectively. Interestingly, the high value of *C_spe_* for the present EDLC assembly is comparable to that reported previously for different polymer electrolytes, as presented in Table 6.

After 1000 cycles, the estimation of efficiency (*η*) of the assembled EDLC is carried out. The efficiency (*η*) is also estimated from the charging time (*t_cha_*) and discharging time (*t_dis_*) using Figure 7 and the equation shown below.

(14) $$\eta =\frac{t_{dis}}{t_{cha}}\times 100$$

**Figure 9:**
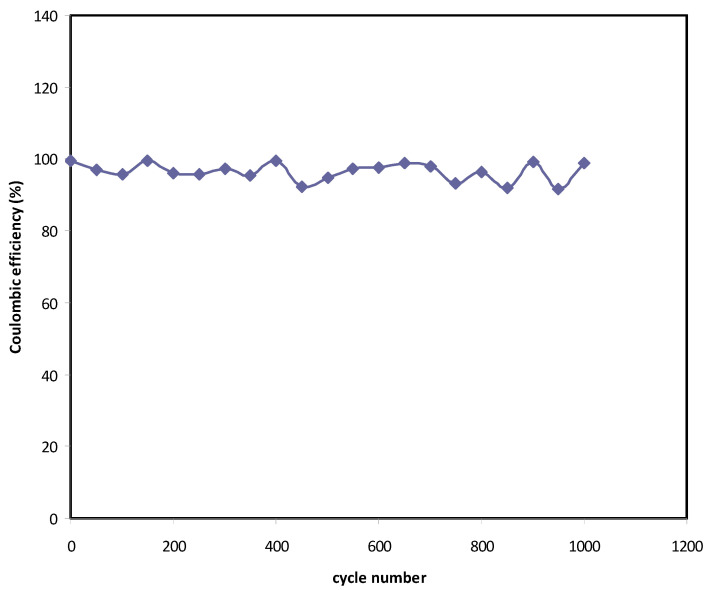
Efficiency of the EDLC throughout the 1000 cycles.

From Figure 9, *η* = 99.5% is obtained at the 1st cycle. Interestingly, beyond the first cycle, the efficiency reached 92% and kept constant up to 1000 cycles. It is worth mentioning that at the steady-state, both the time of charging and discharging are almost the same, which is ideal for a capacitor. The slight reduction of efficiency beyond the first cycle could be due to the development of internal resistance. It is noticed that the decreasing in *η* is harmonized with *C_s_* where it drops from 128 F/g to 119 F/g. Shukur et al. [88] have stated that a convinced EDLC is that which possesses *η* in the 90–95% range. The authors also claimed that to achieve a high efficiency value, it is necessary to have compatibility electrolyte-electrode contact.

It is also critical to identify equivalent series resistance (*R_esr_*) and then evaluate the internal resistance of the EDLC. The mathematical expression of *R_esr_* of the EDLC is shown below.

(15) $$R_{esr}=\;\frac{V_{d}}{i}$$

**Figure 10:**
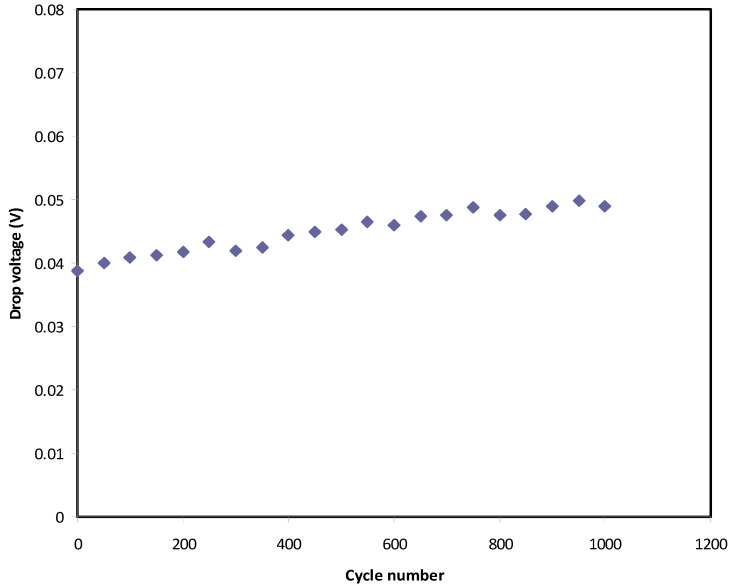
Voltage drop before discharge process for 1000 cycles.

**Figure 11:**
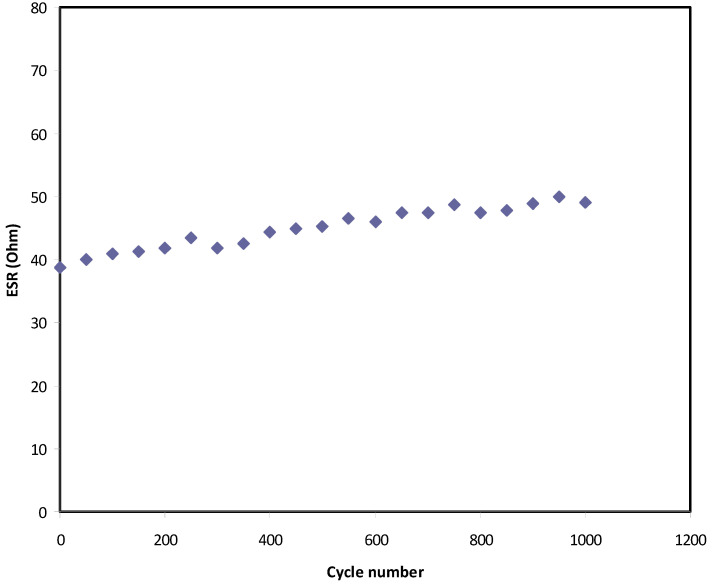
Equivalent series resistance of the EDLC for 1000 cycles.

In Figure 10, a small drop voltage (*V_d_*) occurs before the discharging process. The voltage drop lies in the range 0.039–0.05 V as a consequence of the internal resistance in the EDLC, as shown in Figure 11. It exhibits the *R_esr_* of the EDLC over 1000 cycles. It is seen that the *R_esr_* of the EDLC is in the range of 39 to 50 Ω.

The low value of *R_esr_* indicates the high degree of compatibility between the electrodes and polymer electrolyte. In other words, at the low *R_esr_* value, there is high flux of the ion from the bulk electrolyte region to the electrode surface, thereby, effective double-layer charging occurs [89]. Shuhaimi et al. reported *R_esr_* values of 29 and 64 Ω for a chitosan:κ-carrageenan:NH_4_NO_3_-based EDLC using activated carbon electrodes [90].

Another two crucial and decisive parameters for evaluating an EDLC are energy (*E_den_*) and power (*P_den_*) densities. The former is the energy capacity of the EDLC to be stored, and the latter is the deliver energy or power ability of an EDLC [91]. To calculate both *E_den_* and *P_den_*, the following relationships are used [26,27].

(16) $$E_{den}=\frac{C_{s}V}{2}$$

(17) $$P_{des}=\frac{V^{2}}{4m\left(ESR\right)}$$

**Figure 12:**
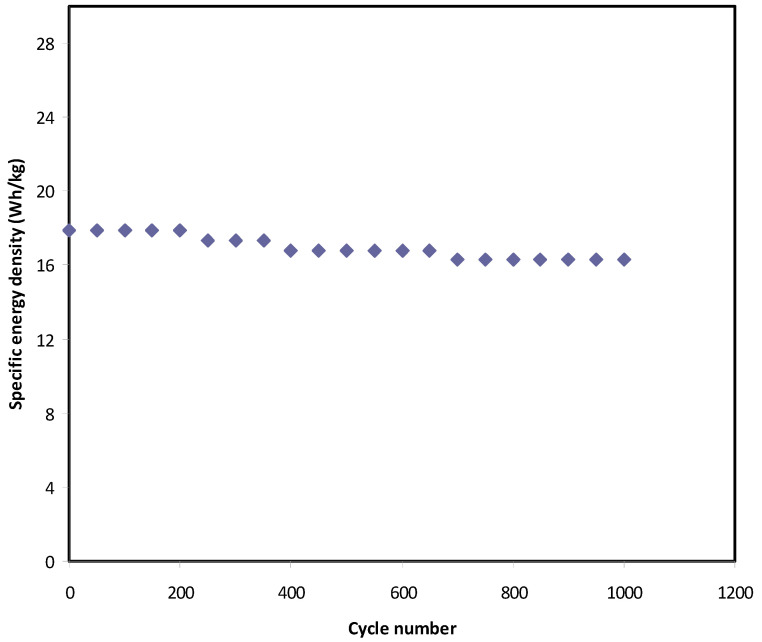
Energy density of the EDLC throughout the 1000 cycles.

**Figure 13:**
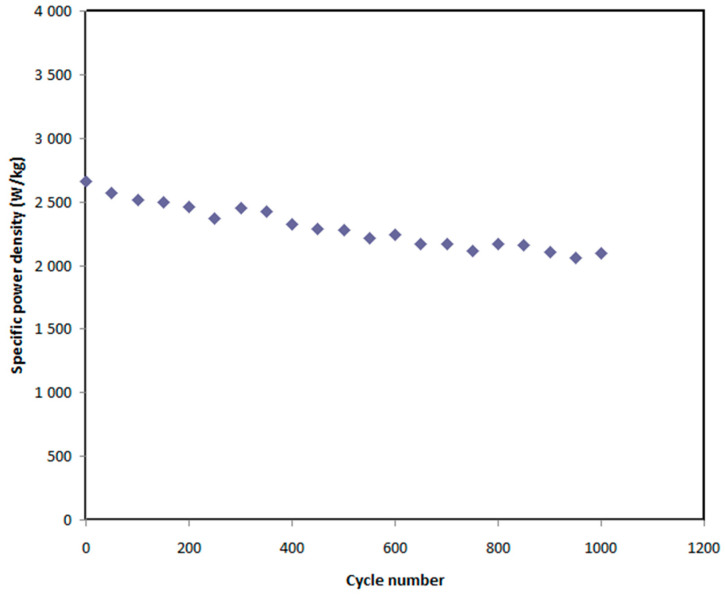
Power density of the EDLC throughout the 1000 cycles.

In this work, the voltage of EDLC assembly was held at 0.9 V. Over 1000 cycles, the *E_den_*, and *P_den_* of the EDLC were estimated as shown in Figure 12 and Figure 13, respectively. At the 1st cycle, the *E_den_* and *P_den_* values are 18 Wh/kg and 2630 W/kg, respectively. It is noticed that both parameters slightly drop to 16.3 Wh/kg and 2300 W/kg, respectively, at the 500th cycle. Beyond the 500th cycle, the energy density and power density become constant with recoding values of 16 Wh/kg and 2050 W/kg, respectively. The high value of *E_den_* obtained in this work is compared to that previously recorded for various polymer electrolytes, as shown in Table 6. For example, the present value of *E_den_* using plasticized polymer electrolyte is higher than the *E_den_* reported for gel SCs using gel polymer electrolyte as documented by Lee et al. [84]. The authors used an ionic liquid-based polymer gel electrolyte in gel SCs, recording an *E_den_* of 15.7 Wh kg^−1^ [84]. However, gel SCs often have higher *E_den_* than solid-state SCs.

In earlier studies [26,27,28,53,54,55], the EDLC-based CS electrolyte as the electrode separator gave lower *E_den_* and *P_den_* than that achieved in this work. This is owing to a lower degree of crystallinity compared to our previous study. It is well-documented that ion conduction is facilitated in amorphous phases [92]. Furthermore, the ion conduction towards the electrode is much enhanced in plasticized electrolytes compared to polymer/salt electrolytes alone. To pinpoint, high EDLC performance can be attributed to the extensive development of double charge layers. It has been documented that the decreasing performance of EDLC, i.e., lowering *C_s_*, *η*, *E_den_* and *P_den_*, results from electrolyte depletion. This phenomenon of electrolyte depletion is due to the rapid charging-discharging process in which free ions in the bulk electrolyte recombine. Under this condition, potential energy developed at the surface of the carbon electrodes decreases enormously [73].

## 4. Conclusions

In summary, proton-conducting solid polymer electrolytes based on CS:NH_4_NO_3_ doped with various quantities of glycerol were used in energy storage electrochemical double-layer capacitor (EDLC) applications. The 40 wt.% of glycerol addition provides relatively high *DC* conductivity of the polymer electrolyte, which is desired. Enrichment of the amorphous domain is also achieved via incorporation of glycerol plasticizer. The maximum conducting plasticized polymer electrolyte possesses a minimum degree of crystallinity. It is also seen that at a relatively high quantity of glycerol, the film is characterized by smoothness and homogeneity of surface morphology. The dominance of ion over electron conductions in the electrolyte was verified as expected. The value of specific capacitance (*C_spe_*) of the EDLC assembly was nearly the same from both the CV response and charge-discharge curve. The voltage window of the electrolyte of interest is satisfactory for utilization in EDLC assembly. The mechanism of charge storing at the interfacial region is rationalized via significant double-layer charging formation.

## Figures and Tables

**Table 1:** The degree of crystallinity calculated from deconvoluted XRD pattern.

Electrolyte	Degree of Crystallinity (%)
Pure CS	15.97
CSNHG2	10.98
CSNHG4	6.93

**Table 2:** *DC* conductivity for plasticized CS:NH_4_NO_3_ systems at room temperature.

Designation	Conductivity (s cm^−1^)
CSNHG1	7.14 × 10^−4^
CSNHG2	1.61 × 10^−3^
CSNHG3	2.14 × 10^−3^
CSNHG4	3.21 × 10^−3^

**Table 3:** The electrical equivalent circuit (EEC) fitting parameters for plasticized electrolyte systems at room temperature.

Sample	*K (F* ^−1^ *)*	*C (F)*
CSNHG1	1.23 × 10^5^	8.13 × 10^−6^
CSNHG2	8.54 × 10^4^	1.17 × 10^−5^
CSNHG3	5.04 × 10^4^	1.98 × 10^−5^
CSNHG4	4.44 × 10^4^	2.25 × 10^−5^

**Table 4:** The values of *ω*, *D*, *µ* and *n* at room temperature.

Sample	*ω* (rad s^−1^)	*D* (cm^2^ s^−1^)	*µ* (cm^2^ V^−1^ s)	*n* (cm^−3^)
CSNHG1	1.70 × 10^6^	1.13 × 10^−7^	4.39 × 10^−6^	1.01 × 10^21^
CSNHG2	1.95 × 10^6^	1.71 × 10^−7^	6.67 × 10^−6^	1.08 × 10^21^
CSNHG3	1.26 × 10^6^	1.86 × 10^−7^	7.26 × 10^−6^	1.84 × 10^21^
CSNHG4	1.13 × 10^6^	2.72 × 10^−7^	1.06 × 10^−5^	1.89 × 10^21^

**Table 5:** Specific capacitance at various sweep rates.

Scan Rate (mV/s)	Capacitance (F/g)
10	91.307
20	77.775
50	53.255
100	31.163

**Table 6:** Specific capacitance, energy density and cycle number of the EDLCs using different polymer electrolytes at room temperature.

Electrolyte Composition	Specific Capacitance (F/g)	Energy Density (Wh/Kg)	Cycle Number	Ref.
PVA:CH_3_COONH_4_:BmImCl	28.36	2.39	500	[81]
MC:Dextran:NH_4_I	79	8.81	100	[82]
PVA:CH_3_COONH_4_:BmImBr	21.89	1.36	500	[83]
EMIM-TFSI:PVDF-HFP	51.8	15.7	3000	[84]
CS:NH_4_F:Zn(II)-complex: glycerol	69.7	7.8	100	[85]
PVA:KOH	112.48	10	1000	[86]
CS:LiCO_2_CH_3_:glycerol	132.8	18.4	700	[87]
**CS:NH_4_NO_3_:glycerol**	**124**	**18**	**1000**	**This work**

## References

[1] Yang Z., Zhang J., Kintner-Meyer M.C.W., Lu X., Choi D., Lemmon J.P., Liu J. (2011). Electrochemical Energy Storage for Green Grid. Chem. Rev..

[2] Powell C.A., Morreale B.D. (2008). Materials Challenges in Advanced Coal Conversion Technologies. MRS Bull..

[3] Arunachalam V., Fleischer E. (2008). The Global Energy Landscape and Materials Innovation. MRS Bull..

[4] Aziz S.B., Brza M., Mohamed P.A., Kadir M., Hamsan M., Abdulwahid R.T., Woo H. (2019). Increase of metallic silver nanoparticles in Chitosan:AgNt based polymer electrolytes incorporated with alumina filler. Results Phys..

[5] Nofal M.M., Aziz S.B., Hadi J.M., Abdulwahid R.T., Dannoun E.M.A., Marif A.S., Al-Zangana S., Zafar Q., Brza M.A., Kadir M.F.Z. (2020). Synthesis of Porous Proton Ion Conducting Solid Polymer Blend Electrolytes Based on PVA: CS Polymers: Structural, Morphological and Electrochemical Properties. Materials.

[6] Suleman M., Deraman M., Othman M.A.R., Omar R., A Hashim M., Basri N.H., Nor N.S.M., Dolah B.N.M., Hanappi M.F.Y.M., Hamdan E. (2016). Electric double-layer capacitors with tea waste derived activated carbon electrodes and plastic crystal based flexible gel polymer electrolytes. J. Physics: Conf. Ser..

[7] Aziz S.B., Hamsan M.H., Brza M.A., Kadir M.F.Z., Muzakir S.K., Abdulwahid R.T. (2020). Effect of glycerol on EDLC characteristics of chitosan:methylcellulose polymer blend electrolytes. J. Mater. Res. Technol..

[8] Zhi J., Yang C., Lin T., Cui H., Wang Z., Zhang H., Huang F. (2016). Flexible all solid state supercapacitor with high energy density employing black titania nanoparticles as a conductive agent. Nanoscale.

[9] Aziz S.B., Karim W.O., Brza M.A., Abdulwahid R.T., Saeed S.R., Al-Zangana S., Kadir M.F.Z. (2019). Ion Transport Study in CS: POZ Based Polymer Membrane Electrolytes Using Trukhan Model. Int. J. Mol. Sci..

[10] Andres B., Dahlström C., Blomquist N., Norgren M., Olin H. (2018). Cellulose binders for electric double-layer capacitor electrodes: The influence of cellulose quality on electrical properties. Mater. Des..

[11] Iro Z.S., Subramani C., Dash S.S. (2016). A Brief Review on Electrode Materials for Supercapacitor. Int. J. Electrochem. Sci..

[12] Brza M., Aziz S.B., Anuar H., Ali F. (2020). Structural, ion transport parameter and electrochemical properties of plasticized polymer composite electrolyte based on PVA: A novel approach to fabricate high performance EDLC devices. Polym. Test..

[13] Brza M., Aziz S.B., Anuar H., Ali F., Hamsan M., Kadir M., Abdulwahid R.T. (2020). Metal framework as a novel approach for the fabrication of electric double layer capacitor device with high energy density using plasticized Poly(vinyl alcohol): Ammonium thiocyanate based polymer electrolyte. Arab. J. Chem..

[14] Inagaki M., Konno H., Tanaike O. (2010). Carbon materials for electrochemical capacitors. J. Power Sources.

[15] Aziz S.B., Brza M.A., Hamsan M.H., Kadir M.F.Z., Muzakir S.K., Abdulwahid R.T. (2020). Effect of ohmic-drop on electrochemical performance of EDLC fabricated from PVA:dextran:NH4I based polymer blend electrolytes. J. Mater. Res. Technol..

[16] Zhang D., Zhang X., Chen Y., Yu P., Wang C., Ma Y. (2011). Enhanced capacitance and rate capability of graphene/polypyrrole composite as electrode material for supercapacitors. J. Power Sources.

[17] Pell W.G., Conway B.E. (2004). Peculiarities and requirements of asymmetric capacitor devices based on combination of capacitor and battery-type electrodes. J. Power Sources.

[18] Yang I., Kim S.-G., Kwon S.H., Lee J.H., Kim M.S., Jung J.C. (2016). Pore size-controlled carbon aerogels for EDLC electrodes in organic electrolytes. Curr. Appl. Phys..

[19] Hou B. (2016). High Specific Surface Area Activated Carbon with Well-Balanced Micro/Mesoporosity for Ultrahigh Supercapacitive Performance. Int. J. Electrochem. Sci..

[20] Chatterjee B., Kulshrestha N., Gupta P. (2015). Electrical properties of starch-PVA biodegradable polymer blend. Phys. Scr..

[21] Karan N.K., Pradhan D.K., Thomas R.H., Natesan B., Katiyar R.S. (2008). Solid polymer electrolytes based on polyethylene oxide and lithium trifluoro- methane sulfonate (PEO–LiCF3SO3): Ionic conductivity and dielectric relaxation. Solid State Ionics.

[22] Aziz S.B. (2013). Li+ ion conduction mechanism in poly (ε-caprolactone)-based polymer electrolyte. Iran. Polym. J..

[23] Dieterich W., Dürr O., Pendzig P., Bunde A., Nitzan A. (1999). Percolation concepts in solid state ionics. Phys. A Stat. Mech. Appl..

[24] Aziz S.B., Abidin Z., Arof A.K. (2010). Effect of silver nanoparticles on the DC conductivity in chitosan–silver triflate polymer electrolyte. Phys. B Condens. Matter.

[25] Hamsan M.H., Aziz S.B., Nofal M.M., Brza M.A., Abdulwahid R.T., Hadi J.M., Karim W.O., Kadir M.F.Z. (2020). Characteristics of EDLC device fabricated from plasticized chitosan:MgCl2 based polymer electrolyte. J. Mater. Res. Technol..

[26] Aziz S.B., Hamsan M.H.H., Nofal M.M.M., San S., Abdulwahid R.T., Saeed S.R.R., Brza M.A., Kadir M.F.Z., Mohammed S.J., Al-Zangana S. (2020). From Cellulose, Shrimp and Crab Shells to Energy Storage EDLC Cells: The Study of Structural and Electrochemical Properties of Proton Conducting Chitosan-Based Biopolymer Blend Electrolytes. Polymers.

[27] Aziz S.B., Brevik I., Hamsan M.H., Brza M.A., Nofal M.M., Abdullah A.M., Rostam S., Al-Zangana S., Muzakir S.K., Kadir M.F.Z. (2020). Compatible Solid Polymer Electrolyte Based on Methyl Cellulose for Energy Storage Application: Structural, Electrical, and Electrochemical Properties. Polymers.

[28] Aziz S.B., Brza M.A., Hamsan H.M., Kadir M.F.Z., Abdulwahid R.T. (2020). Electrochemical characteristics of solid state double-layer capacitor constructed from proton conducting chitosan-based polymer blend electrolytes. Polym. Bull..

[29] Kim J.H., Won J., Kang Y.S. (2004). Olefin-induced dissolution of silver salts physically dispersed in inert polymers and their application to olefin/paraffin separation. J. Membr. Sci..

[30] Hadi J.M., Aziz S.B., Nofal M.M., Hussein S.A., Hamsan M.H., Brza M.A., Abdulwahid R.T., Kadir M.F.Z., Woo H.J. (2020). Electrical, Dielectric Property and Electrochemical Performances of Plasticized Silver Ion-Conducting Chitosan-Based Polymer Nanocomposites. Membranes.

[31] Salleh N.S., Aziz S.B., Aspanut Z., Kadir M. (2016). Electrical impedance and conduction mechanism analysis of biopolymer electrolytes based on methyl cellulose doped with ammonium iodide. Ionics.

[32] Marif A.S., Abdullah R.M., Aziz S.B. (2020). Structural, Morphological, Electrical and Electrochemical Properties of PVA: CS-Based Proton-Conducting Polymer Blend Electrolytes. Membranes.

[33] Li S., Leng D., Li W., Qie L., Dong Z., Cheng Z., Fan Z. (2020). Recent progress in developing Li2S cathodes for Li–S batteries. Energy Storage Mater..

[34] Li S., Fan Z. (2020). Encapsulation methods of sulfur particles for lithium-sulfur batteries: A review. Energy Storage Mater..

[35] Hamsan M.H., Shukur M.F., Aziz S.B., Kadir M.F.Z. (2019). Dextran from Leuconostoc mesenteroides-doped ammonium salt-based green polymer electrolyte. Bull. Mater. Sci..

[36] Hirase R., Higashiyama Y., Mori M., Takahara Y., Yamane C. (2010). Hydrated salts as both solvent and plasticizer for chitosan. Carbohydr. Polym..

[37] Aziz S.B., Rasheed M.A., Ahmed H.M. (2017). Synthesis of Polymer Nanocomposites Based on [Methyl Cellulose]_(1−x)_:(CuS)_x_ (0.02 M ≤ x ≤ 0.08 M) with Desired Optical Band Gaps. Polymers.

[38] Trung T.S., Thein-Han W.W., Qui N.T., Ng C.-H., Stevens W.F. (2006). Functional characteristics of shrimp chitosan and its membranes as affected by the degree of deacetylation. Bioresour. Technol..

[39] Aziz S.B., Abidin Z.H.Z., Kadir M.F.Z. (2015). Innovative method to avoid the reduction of silver ions to silver nanoparticles (Ag+→Ag∘) in silver ion conducting based polymer electrolytes. Phys. Scr..

[40] Bai P., Cao F., Lan X., Zhao F., Ma Y., Zhao C. (2008). Chitosan gel beads immobilized Cu (II) for selective adsorption of amino acids. J. Biochem. Biophys. Methods.

[41] Lu G., Kong L., Sheng B., Wang X., Gong Y., Zhang X. (2007). Degradation of covalently cross-linked carboxymethyl chitosan and its potential application for peripheral nerve regeneration. Eur. Polym. J..

[42] Buraidah M.H., Arof A.K. (2011). Characterization of chitosan/PVA blended electrolyte doped with NH4I. J. Non-Cryst. Solids.

[43] Wan Y., Creber K.A.M., Peppley B., Bui V.T. (2003). Synthesis, characterization and ionic conductive properties of phosphorylated chitosan membranes. Macromol. Chem. Phys..

[44] Osorio-Madrazo A., David L., Trombotto S., Lucas J.-M., Peniche-Covas C., Domard A. (2011). Highly crystalline chitosan produced by multi-steps acid hydrolysis in the solid-state. Carbohydr. Polym..

[45] Aziz S.B. (2016). Role of Dielectric Constant on Ion Transport: Reformulated Arrhenius Equation. Adv. Mater. Sci. Eng..

[46] Aziz S.B., Abidin Z.H.Z. (2015). Ion-transport study in nanocomposite solid polymer electrolytes based on chitosan: Electrical and dielectric analysis. J. Appl. Polym. Sci..

[47] Reddy M.J., Chu P.P. (2002). Ion pair formation and its effect in PEO:Mg solid polymer electrolyte system. J. Power Sources.

[48] Aziz S.B., Abdullah O.G., Rasheed M.A., Ahmed H.M. (2017). Effect of high salt concentration (hsc) on structural, morphological, and electrical characteristics of chitosan based solid polymer electrolytes. Polymers.

[49] Aziz S.B., Abidin Z.H.Z. (2013). Electrical conduction mechanism in solid polymer electrolytes: New concepts to arrhenius equation. J. Soft Matter.

[50] Mobarak N., Ahmad A., Abdullah M., Ramli N., Rahman M.Y.A. (2013). Conductivity enhancement via chemical modification of chitosan based green polymer electrolyte. Electrochim. Acta.

[51] Alves R.D., De Camargo A.S.S., Pawlicka A., Silva M.M. (2016). Luminescent polymer electrolytes based on chitosan and containing europium triflate. J. Rare Earths.

[52] Stavrinidou E., Sessolo M., Winther-Jensen B., Sanaur S., Malliaras G.G. (2014). A physical interpretation of impedance at conducting polymer/electrolyte junctions. AIP Adv..

[53] Aziz S.B., Hamsan M.H., Abdullah R.M., Kadir M.F.Z. (2019). A Promising Polymer Blend Electrolytes Based on Chitosan: Methyl Cellulose for EDLC Application with High Specific Capacitance and Energy Density. Molecules.

[54] Aziz S.B., Abdulwahid R.T., Hamsan M.H., Brza M.A., Abdullah R.M., Kadir M.F.Z., Muzakir S.K. (2019). Structural, Impedance, and EDLC Characteristics of Proton Conducting Chitosan-Based Polymer Blend Electrolytes with High Electrochemical Stability. Molecules.

[55] Aziz S.B., Hamsan M.H., Brza M.A., Kadir M.F.Z., Abdulwahid R.T., Ghareeb H.O., Woo H.J. (2019). Fabrication of energy storage EDLC device based on CS:PEO polymer blend electrolytes with high Li+ ion transference number. Results Phys..

[56] Hamsan M., Aziz S.B., Azha M., Azli A., Shukur M., Yusof Y., Muzakir S., Manan N.S., Kadir M. (2020). Solid-state double layer capacitors and protonic cell fabricated with dextran from Leuconostoc mesenteroides based green polymer electrolyte. Mater. Chem. Phys..

[57] Polu A.R., Kumar R. (2011). Impedance Spectroscopy and FTIR Studies of PEG—Based Polymer Electrolytes. E-J. Chem..

[58] Aziz S.B., Abidin Z.H.Z., Arof A.K. (2010). Influence of silver ion reduction on electrical modulus parameters of solid polymer electrolyte based on chitosan-silver triflate electrolyte membrane. Express Polym. Lett..

[59] Tamilselvi P., Hema M. (2014). Impedance studies of polymer electrolyte based on PVA: PVdF: LiCF3SO3. Int. J. Chemtech Res..

[60] Aziz S.B., Mamand S.M., Saed S.R., Abdullah R.M., Hussein S.A. (2017). New Method for the Development of Plasmonic Metal-Semiconductor Interface Layer: Polymer Composites with Reduced Energy Band Gap. J. Nanomater..

[61] Pradhan D.K., Choudhary R.N.P., Samantaray B.K., Karan N.K., Katiyar R.S. (2007). Effect of Plasticizer on Structural and Electrical Properties of Polymer Nanocompsoite Electrolytes. Int. J. Electrochem. Sci..

[62] Aziz S.B., Abdullah R.M., Kadir M.F.Z., Ahmed H.M. (2019). Non suitability of silver ion conducting polymer electrolytes based on chitosan mediated by barium titanate (BaTiO_3_) for electrochemical device applications. Electrochim. Acta.

[63] Samsudin A.S., Kuan E.C.H., Isa M. (2011). Investigation of the Potential of Proton-Conducting Biopolymer Electrolytes Based Methyl Cellulose-Glycolic Acid. Int. J. Polym. Anal. Charact..

[64] Rani M.S.A., Ahmad A., Mohamed N.S. (2018). Influence of nano-sized fumed silica on physicochemical and electrochemical properties of cellulose derivatives-ionic liquid biopolymer electrolytes. Ionics.

[65] Reddy M.J. (2002). Effect of Mg^2+^ on PEO morphology and conductivity. Solid State Ionics.

[66] Othman L., Isa K.B.M., Osman Z., Yahya R. (2013). Ionic Conductivity, Morphology and Transport Number of Lithium Ions in PMMA Based Gel Polymer Electrolytes. Defect Diffus. Forum.

[67] Vijaya N.N., Selvasekarapandian S.S.S., Malathi J.J., Iwai Y.Y., Nithya H.H., Kawamura J.K.J. (2011). 1H NMR Study on PVP-NH4Cl based- Proton conducting Polymer Electrolyte. Indian J. Appl. Res..

[68] Sampathkumar L., Selvin P.C., Selvasekarapandian S., Perumal P., Chitra R., Muthukrishnan M. (2019). Synthesis and characterization of biopolymer electrolyte based on tamarind seed polysaccharide, lithium perchlorate and ethylene carbonate for electrochemical applications. Ionics.

[69] Monisha S., Mathavan T., Selvasekarapandian S., Benial A.M.F., Latha M.P. (2017). Preparation and characterization of cellulose acetate and lithium nitrate for advanced electrochemical devices. Ionics.

[70] Shukur M.F., Ithnin R., Kadir M.F.Z. (2014). Electrical characterization of corn starch-LiOAc electrolytes and application in electrochemical double layer capacitor. Electrochim. Acta.

[71] Murashko K., Nevstrueva D., Pihlajamäki A., Koiranen T., Pyrhönen J. (2017). Cellulose and activated carbon based flexible electrical double-layer capacitor electrode: Preparation and characterization. Energy.

[72] Hashmi S.A., Latham R.J., Linford R.G., Schlindwein W.S. (1997). Polymer electrolyte based solid state redox supercapacitors with poly (3-methyl thiophene) and polypyrrole conducting polymer electrodes. Ionics.

[73] Liew C.-W., Ramesh S. (2015). Electrical, structural, thermal and electrochemical properties of corn starch-based biopolymer electrolytes. Carbohydr. Polym..

[74] Wang Z., Tammela P., Strømme M., Nyholm L. (2017). Cellulose-based Supercapacitors: Material and Performance Considerations. Adv. Energy Mater..

[75] Varzi A., Balducci A., Passerini S. (2014). Natural Cellulose: A Green Alternative Binder for High Voltage Electrochemical Double Layer Capacitors Containing Ionic Liquid-Based Electrolytes. J. Electrochem. Soc..

[76] Kasprzak D., Stepniak I., Galinski M. (2018). Acetate- and lactate-based ionic liquids: Synthesis, characterisation and electrochemical properties. J. Mol. Liq..

[77] Lim C.-S., Teoh K.H., Liew C.-W., Ramesh S. (2014). Electric double layer capacitor based on activated carbon electrode and biodegradable composite polymer electrolyte. Ionics.

[78] Tripathi M., Tripathi S. (2017). Electrical studies on ionic liquid-based gel polymer electrolyte for its application in EDLCs. Ionics.

[79] Boonen L., Kitzler P., Kasum J. (2018). Processing of aqueous polymer electrolytes for supercapacitors via different industrial application methods. Prog. Org. Coatings.

[80] Łatoszyńska A.A., Taberna P.-L., Simon P., Wieczorek W. (2017). Proton conducting Gel Polymer Electrolytes for supercapacitor applications. Electrochim. Acta.

[81] Liew C.-W., Ramesh S., Arof A. (2014). Good prospect of ionic liquid based-poly(vinyl alcohol) polymer electrolytes for supercapacitors with excellent electrical, electrochemical and thermal properties. Int. J. Hydrogen Energy.

[82] Aziz S.B., Brza M.A., Mishra K., Hamsan M.H., Karim W.O., Abdullah R.M., Kadir M.F.Z., Abdulwahid R.T. (2020). Fabrication of high performance energy storage EDLC device from proton conducting methylcellulose: Dextran polymer blend electrolytes. J. Mater. Res. Technol..

[83] Liew C.-W., Ramesh S., Arof A.K. (2015). Characterization of ionic liquid added poly(vinyl alcohol)-based proton conducting polymer electrolytes and electrochemical studies on the supercapacitors. Int. J. Hydrogen Energy.

[84] Lee J., Kim W., Kim W. (2014). Stretchable Carbon Nanotube/Ion–Gel Supercapacitors with High Durability Realized through Interfacial Microroughness. ACS Appl. Mater. Interfaces.

[85] Asnawi A.S., Aziz S.B., Nofal M.M., Yusof Y.M., Brevik I., Hamsan M.H., Brza M.A., Abdulwahid R.T., Kadir M.F.Z. (2020). Metal Complex as a Novel Approach to Enhance the Amorphous Phase and Improve the EDLC Performance of Plasticized Proton Conducting Chitosan-Based Polymer Electrolyte. Membr..

[86] Yang C.-C., Hsu S.-T., Chien W.-C. (2005). All solid-state electric double-layer capacitors based on alkaline polyvinyl alcohol polymer electrolytes. J. Power Sources.

[87] Asnawi A.S., Aziz S.B., Nofal M.M., Hamsan M.H., Brza M.A., Yusof Y.M., Abdulwahid R.T., Muzakir S.K., Kadir M.F.Z. (2020). Glycerolized Li^+^ Ion Conducting Chitosan-Based Polymer Electrolyte for Energy Storage EDLC Device Applications with Relatively High Energy Density. Polymers.

[88] Shukur M.F., Ithnin R., Kadir M.F.Z. (2014). Protonic Transport Analysis of Starch-Chitosan Blend Based Electrolytes and Application in Electrochemical Device. Mol. Cryst. Liq. Cryst..

[89] Asmara S.N., Kufian M.Z., Majid S.R., Arof A.K. (2011). Preparation and characterization of magnesium ion gel polymer electrolytes for application in electrical double layer capacitors. Electrochim. Acta.

[90] Shuhaimi N.E.A., Alias N.A., Majid S.R., Arof A.K. (2008). Electrical double layer capacitor with proton conducting κ-carrageenan–chitosan electrolytes. Funct. Mater. Lett..

[91] Yang H., Kannappan S., Pandian A.S., Jang J.-H., Lee Y.S., Lu W. (2017). Graphene supercapacitor with both high power and energy density. Nanotechnology.

[92] Pesko D.M., Jung Y., Hasan A.L., Webb M.A., Coates G.W., Miller T.F., Balsara N.P. (2016). Effect of monomer structure on ionic conductivity in a systematic set of polyester electrolytes. Solid State Ionics.

